# A longitudinal study of associations between psychiatric symptoms and disorders and cerebral gray matter volumes in adolescents born very preterm

**DOI:** 10.1186/s12887-017-0793-0

**Published:** 2017-02-01

**Authors:** Violeta L Botellero, Jon Skranes, Knut Jørgen Bjuland, Asta Kristine Håberg, Stian Lydersen, Ann-Mari Brubakk, Marit S Indredavik, Marit Martinussen

**Affiliations:** 1 Department of Laboratory Medicine, Children’s and Women’s Health, Norwegian University of Science and Technology, Medical Technology Research Center, P.O. Box 8905, NO-7491 Trondheim, Norway; 2 Department of Neuroscience, Norwegian University of Science and Technology, Trondheim, Norway; 3 Regional Center for Child and Youth Mental Health and Child Welfare, Norwegian University of Science and Technology, Trondheim, Norway; 4 Department of Pediatrics, Sørlandet Hospital, Arendal, Norway; 5 Department of Medical Imaging, St. Olav’s Hospital, Trondheim University Hospital, Trondheim, Norway; 6 Department of Pediatrics, St. Olav’s Hospital, Trondheim University Hospital, Trondheim, Norway; 7 Department of Child and Adolescent Psychiatry, St. Olav’s Hospital, Trondheim University Hospital, Trondheim, Norway; 8 Department of Gynecology and Obstetrics, St. Olav’s Hospital, Trondheim University Hospital, Trondheim, Norway

**Keywords:** Very preterm, Mental health, Thalamo-cortical system

## Abstract

**Background:**

Being born preterm with very low birthweight (VLBW ≤ 1500 g) poses a risk for cortical and subcortical gray matter (GM) abnormalities, as well as for having more psychiatric problems during childhood and adolescence than term-born individuals. The aim of this study was to investigate the relationship between cortical and subcortical GM volumes and the course of psychiatric disorders during adolescence in VLBW individuals.

**Methods:**

We followed VLBW individuals and term-born controls (birth weight ≥10th percentile) from 15 (VLBW;controls *n* = 40;56) to 19 (*n* = 44;60) years of age. Of these, 30;37 individuals were examined longitudinally. Cortical and subcortical GM volumes were extracted from MRPRAGE images obtained with the same 1.5 T MRI scanner at both time points and analyzed at each time point with the longitudinal stream of the FreeSurfer software package 5.3.0. All participants underwent clinical interviews and were assessed for psychiatric symptoms and diagnosis (Schedule for Affective Disorders and Schizophrenia for School-age Children, Children’s Global Assessment Scale, Attention-Deficit/Hyperactivity Disorder Rating Scale-IV). VLBW adolescents were divided into two groups according to diagnostic status from 15 to 19 years of age: persisting/developing psychiatric diagnosis or healthy/becoming healthy.

**Results:**

Reduction in subcortical GM volume at 15 and 19 years, not including the thalamus, was limited to VLBW adolescents with persisting/developing diagnosis during adolescence, whereas VLBW adolescents in the healthy/becoming healthy group had similar subcortical GM volumes to controls. Moreover, across the entire VLBW group, poorer psychosocial functioning was predicted by smaller subcortical GM volumes at both time points and with reduced GM volume in the thalamus and the parietal and occipital cortex at 15 years. Inattention problems were predicted by smaller GM volumes in the parietal and occipital cortex.

**Conclusions:**

GM volume reductions in the parietal and occipital cortex as well as smaller thalamic and subcortical GM volumes were associated with the higher rates of psychiatric symptoms found across the entire VLBW group. Significantly smaller subcortical GM volumes in VLBW individuals compared with term-born peers might pose a risk for developing and maintaining psychiatric diagnoses during adolescence. Future research should explore the possible role of reduced cortical and subcortical GM volumes in the pathogenesis of psychiatric illness in VLBW adolescents.

**Electronic supplementary material:**

The online version of this article (doi:10.1186/s12887-017-0793-0) contains supplementary material, which is available to authorized users.

## Background

In the past years, an increasing number of studies have reported a significant relationship between being born preterm with very low birth weight (VLBW ≤ 1500 g) and an increased risk of developing psychiatric problems and diagnosis which frequently persist into young adulthood [1–4]. These problems comprise anxiety disorders, attention problems, including attention deficit hyperactivity disorder (ADHD), social difficulties and autism spectrum traits and disorders (ASD) [1–4]. However, the neural basis for this increased risk is not yet fully understood.

Growing evidence from cross-sectional studies suggests that cortical and subcortical gray matter (GM) is especially affected by preterm birth [5–7]. Many studies have related these GM deviations to neurodevelopmental [8–11] and psychiatric problems [12–15] during childhood. However, in a recent MRI meta-analysis, no brain growth rate differences have been found for GM and white matter (WM) volumes between preterm-born children and term-born peers from childhood to adolescence [16]. Even though psychiatric problems may arise any time in life, they commonly appear during adolescence and young adulthood [17–19]. Being born preterm increases the chances of experiencing mental health problems in these risk periods [20, 21]. However, little is known about how these structural changes evolve and their consequences on the development of psychiatric problems that preterm-born individuals experience later in life.

The thalamo-cortical system, which comprises the thalamus, the cerebral cortex and the connecting WM tracts, has been proposed as a major component of the encephalopathy of prematurity [22–24]. Smaller thalamic volume at term-equivalent age has been related to reduced total cerebral cortical volume, suggesting that impaired thalamic growth affects the development of connecting brain structures [24, 25]. Abnormalities in the thalamo-cortical system have been found as a predictor for poor cognitive outcome [26] and impaired social cognition [27]. Volumetric anomalies in thalamo-cortical regions have also been reported in term-born children [28, 29] and adults with ADHD [29, 30].

Moreover, abnormalities in the thalamus shown by surface-based shape analysis have been associated with alterations in the putamen in preterm children, possibly due to disturbed development of shared pre-frontal connectivity [31]. Furthermore, smaller volume of left caudate nucleus has been linked to attention problems in preterm-born male adolescents [32], suggesting that deep GM structures may play a role in attention processes in this population. The thalamus is a key brain structure that connects the brain cortex with the cerebellum, constituting the cerebello-thalamo-cortical pathway, the main efferent cerebellar projection [33]. Disruption in this pathway have been proposed as a major neurobiological mechanism of emotional dysregulation [34]. Increasing evidence points to cerebellar abnormalities in preterm children as a risk factor for developing psychiatric disorders [35, 36]. We have previously reported an association between smaller cerebellar GM volume and persisting/increasing psychiatric symptoms and diagnosis in the same cohort of VLWB adolescents presented in this study [37]. Now, we hypothesize that smaller GM volume of cerebral cortex, thalamus and subcortical structures might be also present in preterm-born adolescents that experience or develop psychiatric problems. Surprisingly, no study so far has examined the impact of cerebral GM deviations in the preterm brain and mental health during adolescence. It is important to study the influence of GM changes on the risk of mental problems on preterm born individuals in order to detect important structure-function relationships and identify possible biomarkers that might help us to spot those at risk and take preventive measures.

The purpose of this study was to investigate the relationship between cerebral GM volumes (cortical GM, subcortical GM and thalamus) and psychiatric disorders and symptoms during adolescence in VLBW individuals, studying both cross-sectional and longitudinal differences. We hypothesized that VLBW adolescents with smaller GM volumes than term-born peers would present higher rates of psychiatric diagnoses and symptoms during adolescence, while having GM volumes similar to controls would be associated with good mental health and/or remission of psychiatric problems. We further hypothesized that there would not be associations between GM volume change from 15 to 19 years and psychiatric symptoms and disorders based on the results from a MRI meta-analysis of de Kiev et al. (2012) [16] and previous findings in our group pointing in the same direction [38]. In order to explore the influence of general cognitive abilities on mental health, we also conducted supplementary analyses including IQ as a covariate and hypothesized that reduced GM volumes would still be an explanatory factor of higher rates of psychiatric symptoms.

## Methods

### Participants

**Fig. 1:**
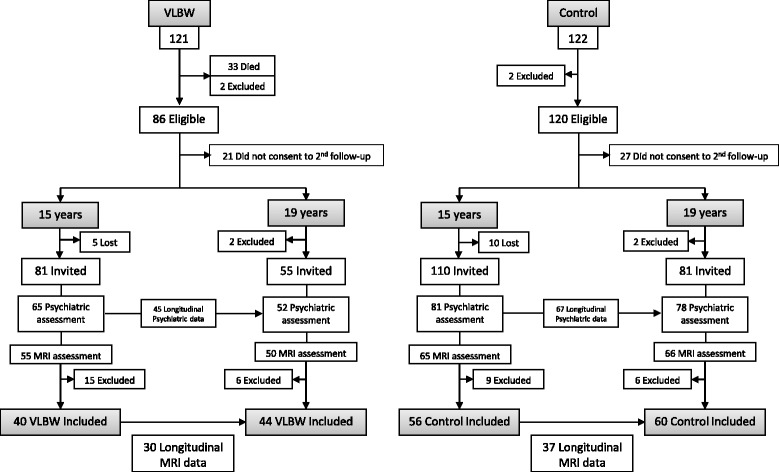
Chart illustrating the composition of the VLBW and control groups at the two measurement points

We studied a cohort of preterm born VLBW (BW ≤ 1500 g; mean birth weight = 1204 g, mean gestational age = 29 weeks) children born in 1986-88 admitted to the neonatal intensive care unit (NICU) at the Trondheim University Hospital (Norway). At the same time, an age-matched group of controls were recruited among term-born children from the same geographical area with birth weight ≥10th percentile for gestational age [38–42] (Fig. 1). For this study, MRI assessments were performed at 15 (Range: VLBW 14y 2mo to 15y 2mo; Controls 14y 1mo to 16y 7mo) and 19 years (Range: VLBW 18y 8mo to 19y 6mo; Controls 18y 8mo to 19y to 8mo). Twelve of the VLBW individuals were born small for gestational age. We obtained structural MPRAGE volumes and psychiatric data of VLBW children and controls at 15 (VLBW;controls *n* = 40;56) and 19 years of age (*n* = 44;60). Of these, 30;37 individuals had longitudinal data. Individuals who had MRIs passing the quality assessment at least at one of the time points were included in the study. MRI images of some participants were discarded due to dental brace artifacts and poor MRI quality due to movement. Two VLBW participants were excluded at both 15 and 19 years due to poor longitudinal surface alignment in the FreeSurfer analyses. At both time points, there were a higher number of participants with psychiatric assessment than MRI scans due to fewer participants giving consent for MRI examination. As a result, some of the participants had longitudinal psychiatric data, but just one MRI assessment. This allowed us to establish diagnostic change also in some participants with only one MRI scan (See Fig. 1 for details).

There were no significant differences between participants (individuals with at least one valid MRI) and non-participants (individuals without MRI) with regard to maternal age at time of birth, birth weight, and gestational age in both groups.

This investigation is the continuation of a previously published study [37]. The clinical data presented here has been earlier reported and some of the tables presented in this article are partial reproductions of our previous work.

The Regional Committee for Medical Research Ethics approved the study protocol (project number: 78-00, May 2000 and 4.2005.2605) and the Data Inspectorate assigned the license for keeping a data register with personal information. Written informed consent was obtained from both adolescents and parents at the 15 years’ assessment, and from the participants at 19 years.

### Psychiatric, cognitive assessment and socio-economic status

The semi-structured diagnostic interview Schedule for Affective Disorders and Schizophrenia for School-age Children (KSADS) [43] was used to obtain psychiatric status of the all participants in the VLBW and the control group. At the first assessment, the interviews were done by two senior clinicians blinded to group status, separately with parents and children. At 19 years, one senior clinician interviewed all participants. Diagnoses were set according to the Diagnostic and Statistical Manual of Mental Disorders, Fourth Edition (DSM-IV) [44] and categorized in three levels according to the KSADS scoring: (I) diagnoses, (II) subclinical diagnoses (≥75% of diagnostic criteria met, but not criteria for full diagnosis), and (III) neither (healthy) [39]. We wanted to study the course of psychiatric disorders. For that, VLBW adolescents were divided into two groups according to diagnostic change from 15 to 19 years of age: (A) persisting/developing diagnosis, (B) healthy/becoming healthy. In the first group, we included those VLBW adolescents who had a psychiatric/subclinical diagnosis at both ages or developed one from 15 to 19 years. In the second group, we included VLBW adolescents who were healthy at both ages or became healthy from 15 to 19 years. This grouping was made post hoc. In our first analyses, we had three VLBW groups (healthy, subclinical diagnosis, diagnosis). Graphs for these previous analyses can be consulted in the Additional file 1.

At the interview, the Children’s Global Assessment Scale (CGAS; scored from 1 to 100) [45] was used to estimate general psychosocial functioning in all participants in the VLBW and the control group. Attention deficit hyperactivity disorder (ADHD) symptoms were evaluated by asking the mothers’ of participants to complete the ADHD Rating Scale-IV (ADHD-RS-IV) Home version [46] for children at the 15-year assessment and the parent-report version for young adults at the 19-year assessment [40, 41].

At 19 years, full IQ was obtained by a senior neuropsychologist [38] with Wechsler Adult Intelligence Scale, 3rd edition (WAIS-III) [47].

Socio-economic status (SES) of the parents was calculated according to the Hollingshead’s Two Factor Index of Social Position, ranging from 1 (low) to 5 (high), based on parents’ education and occupation adapted to today’s categories [48].

### MRI data acquisition and analysis

MRI was performed on the same 1.5 Tesla Siemens Symphony Sonata (Siemens AG, Erlangen, Germany) at St Olav’s University Hospital (Trondheim, Norway) with Quantum gradients (30 mT/m) and a quadrature head coil at 15 and 19 years of age. A structural T1-weighted magnetization prepared rapid acquisition gradient echo (MPRAGE) sequence was acquired with the following specifications: TR = 7.1 ms, TE = 3.45 ms, TI = 1000 ms, flip angle 7^o^, FOV 256 x 256, slab thickness 170 mm, slice thickness 1.33 mm, acquisition matrix 256 x 192 x 128, reconstructed to 256 x 256 x 128, giving a reconstructed voxel resolution of 1 x 1 x 1.33 mm, and acquisition duration of 8.5 min.

The FreeSurfer software package 5.3.0 (http://surfer.nmr.mgh.harvard.edu/) was used for the volumetric parcellation and segmentation. This is an automated method of labeling human structures to extract GM and WM volumes for each participant’s entire brain [49, 50], and parcellating of the cortex of each participant as well as extracting segmentations of subcortical structures [51, 52]. Parcellations of the cortex are automatically corrected for total brain volume differences [51, 52]. In order to avoid segmentation errors, all images were inspected manually and structures with obvious segmentation errors were rejected. No manual adjustments were made to avoid introducing bias and increasing variances into the data set of MRI images.

All images were processed with the longitudinal stream in FreeSurfer 5.3.0 [53–55] to enable longitudinal analyses and to account for unbalanced time points [56]. For each participant, we extracted mean volumes of subcortical GM (caudate nucleus, amygdala, nucleus accumbens, ventral diencephalon, hippocampus and substantia nigra), thalamus, cortical GM volumes for cingulum, frontal lobe, insula, occipital, parietal and temporal lobes, and estimated intracranial volume (eICV).

### Statistical analyses

Data were analyzed using IBM SPSS Statistics version 22 (SPSS, Chicago, IL) and STATA/IC 13.1 (Stata Corporation, College Station, TX, USA). Two-sided *p*-values <0.05 were taken to indicate statistical significance, and 95% confidence intervals (CI) are reported where relevant. All *p*-values were corrected for multiple comparisons following the Benjamini-Hochberg procedure (128 comparisons) [57].

### Background information

Differences in cross-sectional GM volumes between the entire VLBW group and controls were analyzed using a general linear model (GLM), adjusting for age and sex in the analyses of cortical volumes, and age, sex and eICV in the analyses of subcortical structures. Cross-sectional differences between the entire VLBW group and control group on continuous psychiatric variables were analyzed using the Mann-Whitney *U* test and categorical variables and proportions were analyzed by the unconditional z-pooled test (http://www4.stat.ncsu.edu/~boos/exact/) [58]. Perinatal and background information between the two VLBW subgroups on continuous variables were analyzed using the Mann-Whitney *U* test and categorical variables and proportions were analyzed by the unconditional z-pooled test.

### GM volumes and psychiatric data

Group differences in GM volumes at 15 and 19 years of age between the two VLBW subgroups and the control group were calculated with a GLM, including age and sex as covariates in cortical GM analyses, and age, sex and eICV in subcortical GM analyses. Age was included as a covariate in the analyses to account for difference in brain volumes due to age.

In the entire VLBW group, linear regression was used to explore the relationship between GM volumes (independent factor) and psychiatric symptoms assessed with questionnaires (dependent factor) at 15 and 19 years separately. Sex and age were included as covariates in cortical GM analyses. Subcortical GM analyses were also corrected for eICV. Normality of residuals was assessed by visual inspection of Q-Q plots. Missing cases were excluded pairwise.

Longitudinal analyses were done by means of mixed model linear regression, which accounts for missing data, irregular intervals between measures and within person dependence, allowing the combination of cross-sectional and longitudinal data in the same analysis [59]. We calculated the differences in growth trajectories (dependent factor) between the two VLBW subgroups and controls (independent factors), including sex as a covariate in cortical GM analyses, and sex and eICV in subcortical GM analyses. Across the entire VLBW group, we further studied the effect of longitudinal GM volume changes (independent factor) on psychiatric symptoms assessed with questionnaires (dependent factor) including sex as a covariate in cortical GM analyses, and sex and eICV in subcortical GM analyses.

### IQ corrections

In order to explore the influence of general cognitive abilities on the relationship between GM volumes and psychiatric symptoms, the analyses were further adjusted for full IQ obtained at 19 years. As IQ can be both a risk factor for psychiatric problems and affected by them, the results are presented before corrections to avoid shadowing any direct relationship between brain abnormalities and psychiatric symptoms [60].

## Results

### Psychiatric and MRI findings

Neonatal and socio-demographic variables are displayed in Table 1. These data have been previously published [37]. There were no differences in any of the variables between the cross-sectional and longitudinal data within the study groups. Birth weight and gestational age differed by design between the VLBW and the control group. The VLBW group also had lower IQ scores. There were no differences in socio-economic status between the groups, except for SES class 1, where we found a higher percentage of SES class 1 in VLBW individuals than in controls.

**Table 1:** Participants’ neonatal and socio-demographic details

	Assessed at 15 years		Assessed 19 at years			Assessed at both time points	
	VLBW	Control	VLBW	Control		VLBW	Control
Number of participants	40	56	44	60		30	37
Males (%)	18 (45)	21 (37)	18 (41)	25 (42)		11 (37)	14 (38)
Background information							
Birthweight (grams) M (SD)	**1204** (236)***	3713 (500)	**1212** (234)***	3698 (501)		**1223** (250)***	3766 (544)
Gestational age (weeks) M﻿ (SD)	**29.18** (2.65)***	39.61 (1.15)	**29.25** (2.54)***	39.72 (1.27)		**29.43** (2.60)***	39.51 (1.17)
Age (years-months) M (SD)	15-2 (0-6)	15-5 (0-5)	19-7 (0-7)	19-8 (0-6)	Time 1	15-2 (0-6)	15-5 (0-5)
					Time 2	19-9 (0-8)	19-7 (0-6)
IQ M (SD)			**89.00** (12.54)***	99.85 (10.62)		**86.33** (13.52)***	100.14 (11.03)
SES (1 – 5) M (SD)	3.15 (1.25)	3.59 (1.04)	3.39 (1.38)	3.70 (0.95)		3.27 (1.33)	3.65 (0.92)
SES class 1 n (%)	**5** (12) **	0 (0)	**6** (15) *	1 (2)		**4** (13) *	0 (0)
SES class 2 n (%)	7 (17)	10 (18)	5 (12)	4 (7)		5 (17)	4 (11)
SES class 3 n (%)	11 (28)	16 (29)	7 (17)	17 (32)		6 (20)	12 (32)
SES class 4 n (%)	11 (28)	17 (30)	13 (32)	21 (39)		9 (30)	14 (38)
SES class 5 n (%)	6 (15)	13 (23)	10 (24)	11 (20)		6 (20)	7 (19)

*p ≤ 0.05, **p ≤ 0.01, ***p ≤ 0.001 (VLBW versus controls). Significant results marked bold. Linear regression adjusted for age and sex for normal distributed data, else the Mann–Whitney *U*-test

The unconditional z-pooled test was used to analyze differences in proportions between groups

*Abbreviations*: *IQ* Intelligence quotient, *M* Mean, *SD* standard deviation, *SES* socio-economic status, *VLBW* very low birth weight (birth weight ≤ 1500)

A version of this table has been previously published by our group [37]. In this new version we have included detailed data regarding SES class results

Brain volumes and clinical findings are given in Table 2. Brain volumes of cingulum, frontal, occipital, parietal, and temporal cortices, insula, thalamus and subcortical GM were, at both ages, smaller in the VLBW than in the control group. Estimated psychosocial functioning was lower (lower CGAS scores) and ADHD symptoms were more pronounced (higher scores on the Inattention subscale) at both ages in the VLBW group than in the control group. There were also, at both ages, higher frequencies of any psychiatric disorder in the VLBW group, in particular, ADHD diagnoses at both ages and anxiety disorders at 19 years. Fewer VLBW adolescents than controls were or became healthy during the study period, and more VLBW adolescents than controls had or developed psychiatric problems. At the 15-year assessment, Intra Class Correlation between the two interviewers was 0.91 for any diagnosis/subclinical diagnosis. At 19 years, all the interviews were conducted by the same clinician, therefore, inter-rater reliability tests were not performed.

**Table 2:** Brain volumes and psychiatric outcome in VLBW participants and controls

	15 years		19 years	
	VLBW (*n* = 40)	Control (*n* = 56)	VLBW (*n* = 44)	Control (*n* = 60)
Brain volumes (ml)				
Cortical gray matter				
Cingulum M (SD)	**21.85** (3.21)***	24.14 (2.93)	**20.64** (3.16)***	23.07 (2.79)
Frontal cortex M (SD)	**188.21** (28.71)**	201.80 (16.71)	**117.39** (28.83)***	190.93 (16.94)
Insula M (SD)	**13.01** (2.10)***	14.37 (1.40)	**12.79** (2.25)***	13.83 (1.39)
Occipital cortex M (SD)	**48.65** (6.65)*	50.94 (4.48)	**46.79** (6.66)*	49.08 (4.52)
Parietal cortex M (SD)	**117.86** (16.57)***	133.58 (11.74)	**110.68** (14.41)***	124.54 (11.04)
Temporal cortex M (SD)	**110.80** (16.92)***	125.72 (12.40)	**107.64** (16.65)***	120.15 (11.65)
Thalamus M (SD)	**13.15** (1.83)***	15.35 (1.27)	**13.24** (1.81)***	15.30 (1.32)
Subcortical gray matter M (SD)	**43.79** (4.93)***	49.72 (3.55)	**44.12** (5.03)*	48.22 (3.96)
Psychiatric results				
CGAS M (SD)	**71.73** (14.48)***	86.96 (6.75)	**79.05** (12.75)**	85.78 (7.69)
ADHD-RS-IV - mother				
Hyperactivity M (SD)	2.78 (3.71)	1.43 (1.78)	2.90 (4.29)	1.34 (1.67)
Inattention M (SD)	**6.39** (5.11)***	2.51 (2.81)	**5.45** (5.58)**	1.76 (1.98)
Any psychiatric diagnosis n (%)	**12** (30)**	3 (5)	**11** (25)**	4 (7)
Anxiety disorders^a^ n (%)	5 (13)	2 (4)	**7**(16)**	1 (2)
ADHD n (%)	**3** (8)*	0 (0)	**4** (9)*	0 (0)
Other^b^ n (%)	4 (10)	1 (2)	0 (0)	3 (5)
Any Subclinical diagnosis n (%)	**11** (28)***	1 (2)	5 (11)	6 (10)
Anxiety disorders^a^ n (%)	3 (8)	1 (2)	4 (9)	2 (3)
ADHD n (%)	**8** (20)***	0 (0)	1 (2)	3 (5)
Other^b^ n (%)	0 (0)	0 (0)	0 (0)	1 (2)
Diagnostic status				
Healthy/Becoming healthy n (%)	**22** (55)**	46 (82)	**25** (61)**	50 (85)
Persisting/Developing diagnosis n (%)	**18** (45)**	10 (18)	**16** (39)*	9 (15)

*p ≤ 0.05, **p ≤ 0.01, ***p ≤ 0.001 (VLBW versus controls). Significant results marked bold. Linear regression adjusted for age and sex for normal distributed data, else the Mann–Whitney *U*-test. The unconditional z-pooled test was used to analyze differences in proportions between groups. Subcortical brain volumes were further adjusted for estimated intracranial volume

*Abbreviations*: *ADHD-RS-IV* Attention-Deficit/Hyperactivity Disorder Rating Scale, *CGAS* children’s global assessment scale, *SD* standard deviation, *VLBW* very low birth weight (birth weight ≤ 1500)

^**a**^Anxiety disorders: separation anxiety disorder, generalized anxiety disorder, social phobia, or specific phobia

^**b**^Other: Asperger’s disorder, depressive disorder, adjustment disorder, elimination disorder, post-traumatic stress disorder, stuttering, tic disorder. None had manic or bipolar, psychotic, or eating disorder

A version of this table has been previously published by our group [37]. In this new version we have included new data regarding gray matter volume results

VLBW adolescents who had or developed psychiatric problems had significantly lower birth weight, lower 1-min Apgar score and lower IQ at 19 years than VLBW adolescents who were or became healthy. However, they did not differ in z-score birth weight (individual standard deviation scores for birth weight, representing the deviation from the mean weight for sex, gestational age, and singleton [61] or multiple births [62], a measure of intrauterine growth failure) gestational age, head circumference at birth, days before regaining birth weight, days on ventilator, days in the NICU, 5-min Apgar score, socio-economic status or mother’s age (Table 3).

**Table 3:** Perinatal and background information in VLBW participants according to diagnostic status during adolescence

	Persisting/Developing diagnosis (*n* = 24)	Healthy/Becoming healthy (*n* = 30)
Male n (%)	10 (41.7)	14 (46.7)
Birth weight (grams) M (SD)	**1096.25** (264.41)**	**1269.30** (159.40)**
z-score weight M (SD)	-0.82 (1.50)	-0.52 (1.40)
Gestational age (weeks) M (SD)	28.80 (2.93)	29.67 (2.59)
Head circumference (cm) M (SD)	26.52 (2.51)	26.64 (1.64)
Days before regained weight M (SD)	16.11 (9.38)	16.62 (7.82)
Days on ventilator M (SD)	9.45 (17.52)	2.22 (3.80)
Days in NICU M (SD)	92.15 (84.59)	58.89 (21.92)
Apgar 1 min M (SD)	**5.71** (2.70)**	**7.65** (1.38)**
Apgar 5 min M (SD)	7.89 (2.36)	9.04 (0.87)
IQ 19 years M (SD)	**80.00** (71.59)**	**93.04** (9.40)**
Socio-economic status M (SD)	3.09 (1.37)	3.37 (1.27)
SES class 1 n (%)	4 (19)	3 (10)
SES class 2 n (%)	3 (14)	4 (14)
SES class 3 n (%)	6 (29)	6 (21)
SES class 4 n (%)	4 (19)	10 (34)
SES class 5 n (%)	4 (19)	6 (21)
Mother’s age (years) M (SD)	42.95 (4.59)	43.37 (4.96)

*p ≤ 0.05, **p ≤ 0.01, ***p ≤ 0.001 (Persisting/increasing VLBW versus Healthy/decreasing VLBW). Significant results marked bold. Mann–Whitney *U*-test. Z-score weight: Standard deviation score of weight in relation to gestational age and gender. *Abbreviations*: *IQ* Intelligence quotient, *NICU* Neonatal Intensive Care Unit, *SD* Standard deviation, *VLBW* Very low birth weight (birth weight ≤ 1500)

### Relationship between GM volumes and psychiatric data

#### GM volume and diagnostic status during adolescence

**Fig. 2:**
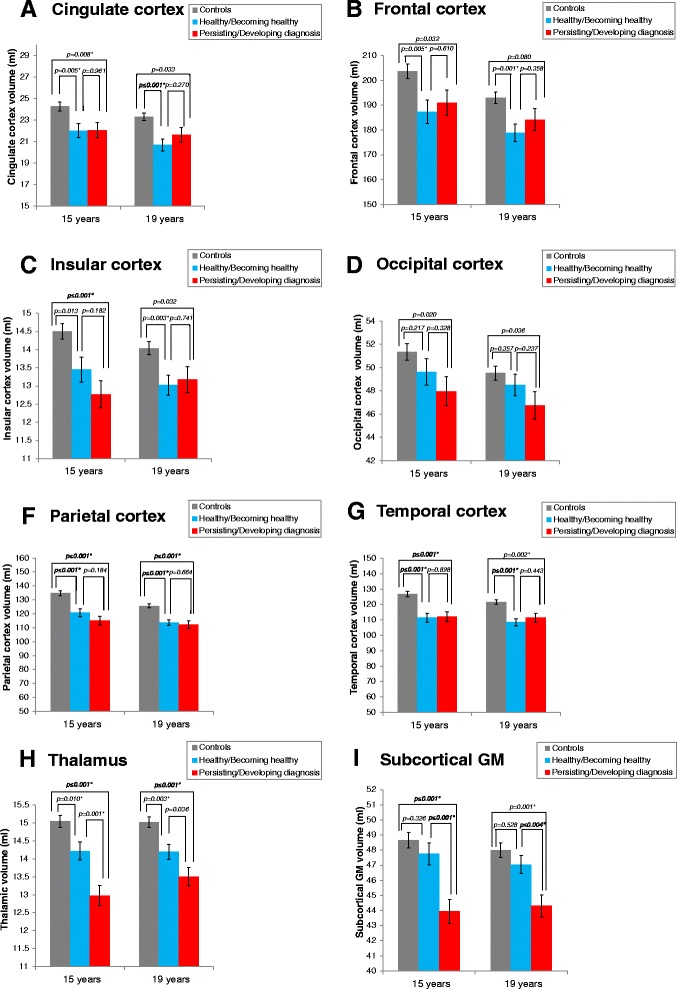
Brain volumetric differences between the two VLBW subgroups and controls at 15 and 19 years. The two VLBW diagnostic subgroups presented volume reductions in several cortices **a**-**g** and thalamus **h** compared with the control group. Subcortical GM reductions **i** were limited to the persisting/developing diagnosis VLBW subgroup. Results adjusted for age and sex. Subcortical structures adjusted for estimated intracranial volume. Abbreviations: GM: Gray matter. * Significant results after adjusting for multiple testing

GM volumes in the two VLBW subgroups and controls are displayed in Fig. 2. The two VLBW subgroups tended to have smaller volumes than controls in all cortical areas at both ages, but not all differences reached statistical significance. The volume of cingulate cortex was smaller in both VLBW subgroups at 15 years, whereas at 19 years of age, this was found only in the healthy/becoming healthy VLBW subgroup compared with the control group. The healthy/becoming healthy VLBW subgroup had smaller volume of frontal cortex than the control group at both ages. Insula volume was smaller in the VLBW subgroup with persisting/developing diagnosis than in the control group at 15 years of age, and smaller than controls in the healthy/becoming VLBW subgroup at 19 years. Both VLBW subgroups had, at both ages, smaller parietal and temporal cortical volumes than controls. There were no differences in cortical volumes between the two VLBW subgroups (Fig. 2a-g).

Both VLBW subgroups had, at both ages smaller thalamic volume than controls at both ages (15 years: Persisting/Developing diagnosis vs controls: MD = -2.068, SE = 0.330, (-2.723 to -1.413), p ≤ 0.001; Healthy/Becoming healthy vs controls: MD -0.829, SE = 0.317, (-1.459 to -0.200), *p* = 0.010. 19 years: Persisting/Developing diagnosis vs controls: MD = -1.516, SE = 0.300, (-2.112 to -0.920), *p* ≤ 0.001; Healthy/Becoming healthy vs controls: MD = -0.826, SE = 0.266, (-1.355 to -0.297), *p* = 0.003). Thalamic volume was smaller in the persisting/developing diagnosis VLBW subgroup compared with the healthy/becoming healthy VLBW subgroup at 15 years (MD = -1.239, SE = 0.363, (-1.961 to -0.517), *p* = 0.001) (Fig. 2h).

Subcortical volumes were smaller only in the persisting/developing diagnosis VLBW subgroup compared with the control group at both ages (15 years: MD = -4.719, SE = 0.948, (-6.602 to -2.837), *p* ≤ 0.001. 19 years: MD = -3.213, SE = 0.856, (-4.913 to -1.513), *p* ≤ 0.001), whereas no differences were found between the healthy/becoming healthy VLBW subgroup and the control group. Smaller subcortical GM volumes were found in the persisting/developing diagnosis VLBW subgroup compared with the healthy/becoming healthy VLBW subgroup at both ages (15 years: MD = -3.820, SE = 1.045, (-5.895 to -1.744), *p* ≤ 0.001; 19 years: MD = -2.731, SE = 0.926, (-4.569 to -0.893), *p* = 0.004) (Fig. 2i).

After correcting for IQ, both VLBW subgroups had persistent smaller volume of parietal cortex than controls at both time points. The healthy/becoming healthy VLBW subgroup had smaller cingulate and temporal volumes at both ages and smaller frontal cortical volume at 19 years than the control group. Thalamic volumes were smaller in the VLBW subgroup with persisting/developing diagnosis than in controls at both ages. Detailed results of differences in brain volumes between the two VLBW subgroups and controls before and after corrections for IQ are provided in Additional files 2 and 3: Appendix 1 A-B respectively.

**Fig. 3:**
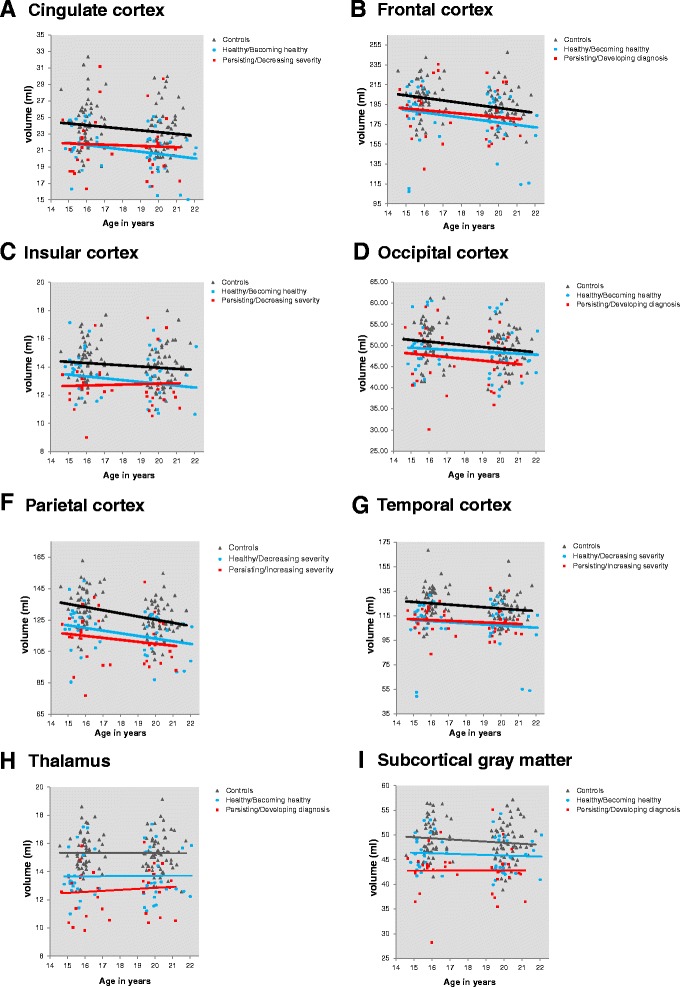
Brain developmental differences between the two VLBW groups and controls from 15 to 19 years. There were not any differences in cortical **a**-**g** and subcortical **h**-**i** volume growth between the two VLBW subgroups and controls. Results adjusted for sex. Subcortical structures adjusted for estimated intracranial volume. Abbreviations: GM: Gray matter; VLBW: Very low birth weight

Mixed linear model analyses revealed that there were no differences in GM volume growth rate in the brain cortex, thalamus and subcortical GM between the two VLBW subgroups and controls (Fig. 3). Detailed results are provided in Additional files 4 and 5: Appendix 2A-B.

### GM volume and psychosocial functioning

**Fig. 4:**
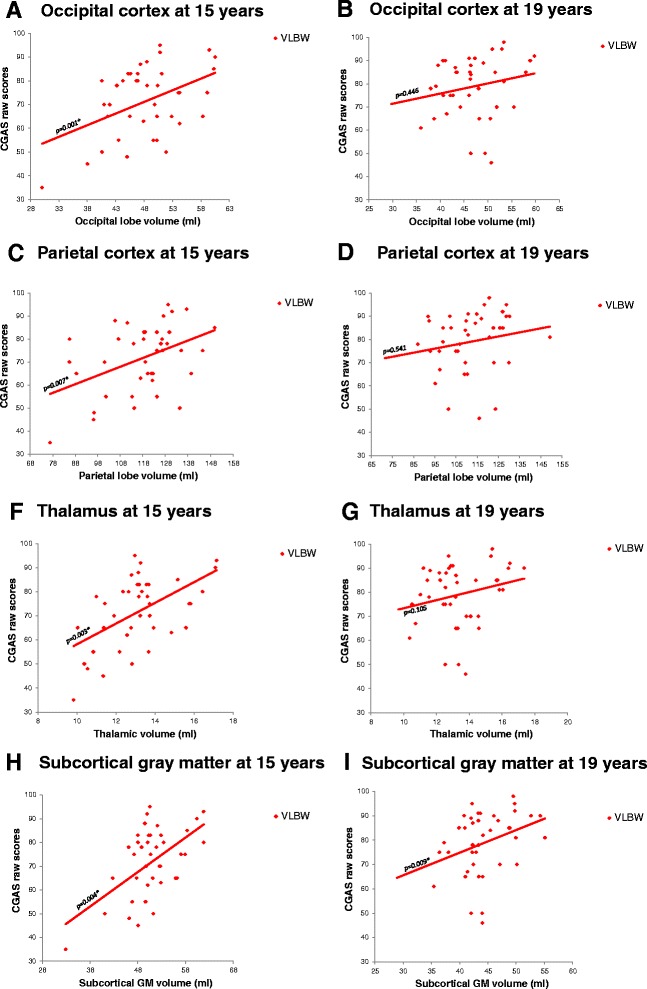
Relationships between brain volumes and psychosocial functioning in the VLBW group. Occipital **a**-**b** and parietal volume **c**-**d** reductions at 15 years predicted poorer psychosocial functioning in the VLBW group. Smaller volumes of thalamus **f**-**g** and subcortical GM **h**-**i** were associated with poorer psychosocial functioning in the VLBW group at both ages. Results adjusted for age and sex. Subcortical structures adjusted for estimated intracranial volume. Abbreviations: CGAS: Children’s Global Assessment Scale; GM: Gray matter; VLBW: Very low birth weight. * Significant results after adjusting for multiple testing

At 15 years of age, smaller volumes of occipital and parietal cortex and of thalamus predicted lower scores in general psychosocial functioning across the entire VLBW group (Occipital cortex: B = 1.107 (0.465 to 1.750), *p* ≤ 0.001; Parietal cortex: B = 0.366 (0.109 to 0.622), *p* = 0.007; Thalamus: B = 3.990 (1.457 to 6.523), *p* = 0.003). Smaller subcortical GM volumes predicted lower psychosocial functioning (CGAS scores) at both 15 and 19 years in the VLBW group (15 years: B = 1.441 (0.505 to 2.377), *p* = 0.004; 19 years: B = 1.454 (0.391 to 2.517), *p* = 0.009) (Fig. 4). After correcting for IQ, occipital and parietal cortex volumes still predicted lower scores in general psychosocial functioning at 15 years, but the volumes exerting the effect were smaller. Detailed results before and after corrections for IQ are provided in Additional files 6 and 7: Appendix 3 A-B.

We did not find any associations between GM volume growth rate in the brain cortex, thalamus and subcortical GM and CGAS scores in the VLBW group (Additional files 8 and 9: Appendix 4 A-B).

### GM volume and ADHD

**Fig. 5:**
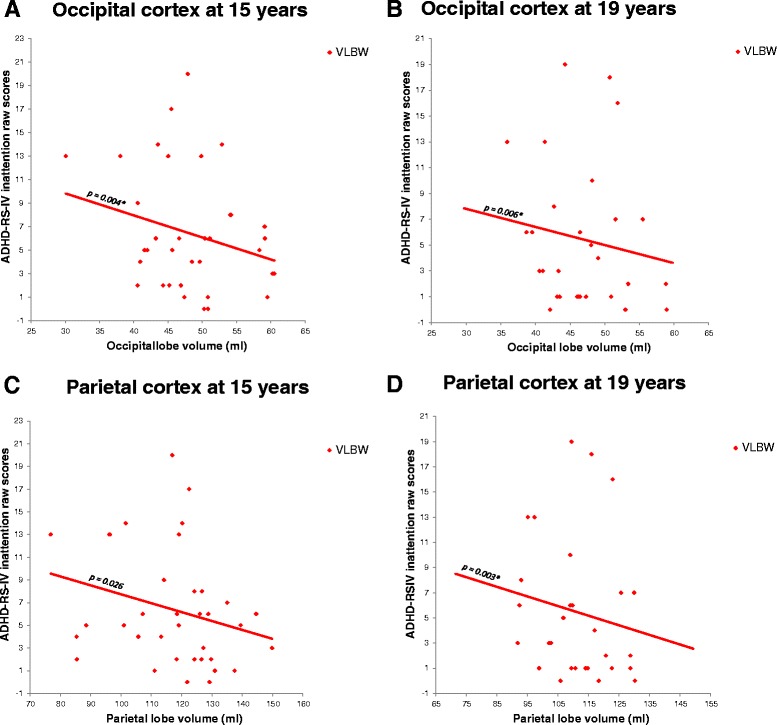
Relationships between brain volumes and inattention in the VLBW group. Smaller volumes in occipital **a**-**b** and parietal **c**-**d** cortices predicted higher inattention scores in the VLBW group at 19 years. Results adjusted for age and sex. Subcortical structures adjusted for estimated intracranial volume. Abbreviations: GM: Gray matter; VLBW: Very low birth weight. * Significant results after adjusting for multiple testing

Smaller volumes of occipital and parietal cortex predicted higher inattention scores in the VLBW group at both ages, although not all differences survived corrections for multiple comparisons (15 years, occipital cortex: B = -0.356 (-0.593 to -0.119), *p* = 0.004; 19 years, occipital cortex: B = -0.408 (-0.689 to -0.127), *p* = 0.006; 19 years, parietal cortex: B = -0.202 (-0.331 to -0.072), *p* = 0.003) (Fig. 5). After correcting for IQ, smaller volumes of occipital and parietal cortex predicted higher hyperactivity scores at 15 years. Detailed results before and after corrections for IQ are provided in Additional files 6 and 7: Appendix 3 A-B.

We did not find any associations between GM volume growth rate in the brain cortex, thalamus and subcortical GM and ADHD-RS mother-report scores in the VLBW group (Additional files 8 and 9: Appendix 4 A-B).

## Discussion

We have followed a cohort of VLBW adolescents and controls from 15 to 19 years of age in order to study the associations between cortical, thalamic and subcortical GM volume development and mental health status and course. Our main result was a finding of sustained smaller subcortical GM volume, not restricted to the thalamus, during adolescence in the VLBW subgroup with persisting/developing psychiatric diagnosis compared with both the control group and the VLBW healthy/becoming healthy subgroup. However, no difference in subcortical GM volume was found between the VLBW healthy/becoming healthy subgroup and controls at 15 or 19 years of age (Fig. 2i). Across the entire VLBW group, lower psychosocial functioning was predicted by smaller thalamus, parietal and occipital cortices at 15 years, and by smaller subcortical GM volume at both time points. Inattention symptoms were predicted by smaller GM volumes in the occipital and parietal cortex (Fig. 5). We did not find any differences in volume growth between the two VLBW subgroups and controls (Fig. 3).

Subcortical GM, and especially the thalamus, appears particularly vulnerable to preterm birth, even in the absence of acute focal WM injury [63, 64]. There is considerable evidence that smaller volumes of thalamus and deep GM nuclei in children born preterm are associated with poorer cognitive performance in childhood and adolescence [9, 10, 26, 65–67]. However, little is known about its impact on mental health. There is one study suggesting that alterations in the cortico-basal ganglia-thalamo-cortical loop connections and the short cortico-cortical connections following preterm birth might contribute to poorer prosocial behavior, recognition of social context, and simultaneous information processing in childhood [27]. Volumetric abnormalities in the hippocampus, amygdala, and putamen from early to mid-adolescence have been also linked to onset of depression during this important period of life [68]. In line with these investigations, our results suggest that structural alterations in subcortical structures, not restricted to the thalamus, following preterm birth might be a risk factor for developing and maintaining psychiatric problems during adolescence.

Our results also suggest that smaller GM volumes in subcortical nuclei, thalamus and occipital and parietal cortex during adolescence are important explanatory factors for higher inattention scores and lower psychosocial functioning in VLBW adolescents. It has been suggested that attention problems in very preterm born children might be related to abnormalities in the fronto-parietal network, which is implicated in orienting, alerting and executive attention [69]. Traditionally, the occipital lobe has not been associated with attention problems. However, Ahrendts et al. reported volume reduction in the visual cortex in term-born adults diagnosed with ADHD [70], suggesting that this region may be of interest in ADHD due to its involvement in visual information processing [71]. Our results support Ahrendts et al. [70] results, suggesting that different mechanisms might be involved in the development of ADHD in preterm-born children, distinct from full-term children. Interestingly, reduced GM volumes of subcortical structures and cortical areas, including the parietal and occipital cortex, measured at term equivalent age in preterm-born children have been linked to ADHD [12, 13], social problems [14] and ASD [15] during childhood, suggesting that brain growth deviations in these areas occurring in the neonatal period may persist into adolescence and adulthood [16, 72, 73] and have an impact on mental health.

We did not find any differences in volume growth between the two VLBW diagnostic subgroups and controls. We have previously reported similar results between the entire VLBW group and controls without differentiating according to psychiatric status [38]. Our findings of similar brain growth rate during this period are supported by a meta-analysis of de Kiev et al. (2012) who found that brain growth trajectories did not differ between preterm and term-born children from 8 to 18 years [16]. However, in those born preterm, smaller GM volumes seem to be present from birth to young adulthood [16, 38, 42, 72–74], especially of deep GM nuclei [38, 63–66, 73–78]. Our results suggest that brain volumes might be even smaller in those VLBW individuals who develop or maintain psychiatric problems during adolescence compared with those VLBW adolescents who are or become healthy in this period.

Interestingly, several structural MRI studies have reported deviant brain growth in cortical GM [11, 79, 80], subcortical GM [24, 63, 64, 79, 81, 82], WM microstructure [10, 79, 83–87] and regional brain growth [10, 24, 79, 88] around term-equivalent age. These growth deviations occurring after birth have been related to deficits in cognition [8–11, 87], motor performance [8, 87], visual motor integration [9], language [9] and mental health [12–15] during childhood. We found differences in SES class 1 between the VBLW group and controls. These results are in line with previous studies that indicate that lower SES has an impact in birth weight [89]. However, we did not find differences in any of the SES classes between the two VLBW subgroups, suggesting that SES might not be an explanatory factor for the higher rates of psychiatric symptoms in VLBW individuals. We found that the VLBW subgroup with persisting/developing psychiatric diagnosis had significant lower birth weight and 1-min Apgar scores than the subjects in the VLBW subgroup who were/became healthy during adolescence. Thus, we speculate that the most fragile newborns might have had deviant brain development in the neonatal period, which already at that time could have been a predictor for mental health development.

There is evidence that reduced connectivity in the thalamo-cortical system is associated with poorer social reasoning skills, more peer problems and worse prosocial behavior in preterm-born children at the age of six [27]. However, others point to the cerebellum as a critical structure involved in the higher prevalence of psychiatric disorders in these children [35, 36]. We have previously reported an association between persistent smaller cerebellar GM and WM volumes during adolescence and psychiatric symptoms and disorders and psychosocial functioning in this VLBW group. Our aim was to study the relationship between cerebellar volumes and psychiatric diagnoses and symptoms in VLBW adolescents. We found that VLBW adolescents with persisting/developing diagnosis had smaller cerebellar GM and WM volumes than controls and healthy/becoming healthy VLBW adolescents [37]. The cerebello-thalamo-cortical system along with deep GM nuclei may be especially vulnerable to damage during the third trimester of gestation, during which several developmental events take place, involving axons, pre-myelinatingolig odendrocytes (pre-OLs), subplate neurons, microglia, and cell migration from subventricular zone [6, 90]. It has also been suggested that deep GM and cerebellar abnormalities might be caused by problems with the microstructural organization of large WM pathways, such as thalamo-cortical, fronto-striatal, and fronto-cerebellar tracts, connecting these structures with the cortex [10, 91, 92]. Future research should focus on this system to elucidate its implication in mental health disorders in VLBW individuals.

It is also of interest to evaluate the influence of general cognitive abilities on the relationship between GM volumes and psychiatric symptoms, psychosocial functioning and ADHD symptoms. Recent research suggests that impaired executive function (i.e., inhibition, working memory, and cognitive flexibility) is a core feature in many mental illnesses [93]. VLBW children commonly experience higher rates of both cognitive and psychiatric problems than their term-born peers [94]. Autistic and ADHD symptoms have been found to correlate with cognitive function in VLBW children [95, 96]. Still, the background for this correlation is not fully understood yet [97, 98]. One possibility is that cognitive outcomes might be affected by attention problems that interfere during cognitive evaluation [99]. Another possibility is that cognitive skills might be affected by the same brain mechanisms which affect mental health problems in these children. Poor cognitive performance in VLBW individuals has been associated with abnormalities in extensive areas of the cerebral cortex and subcortical structures [9, 38, 72, 76, 100]. Interestingly, Ball et al. found thalamo-cortical structural connectivity at term to be a strong predictor of cognitive scores at 2 years in children born preterm [26]. These brain areas have also been related to psychiatric symptoms in the preterm-born population [12, 13, 27, 32, 101]. After correcting for IQ, we found that smaller volumes in the parietal cortex and thalamus at both 15 and 19 years in the persisting/developing diagnosis VLBW subgroup were still significant, suggesting that psychiatric problems in VLBW individuals are not only explained by deficits in cognition, although they might be related and share similar brain correlates. More research is necessary to explain how cognitive and psychiatric problems relate to each other and what the neural basis for the two is.

In this study, cortical and subcortical segmentations were calculated using FreeSurfer 5.3.0, a well-known and reliable automated MR segmentation method to measure GM volumes (http://surfer.nmr.mgh.harvard.edu/). FreeSurfer has proved test-retest reproducibility across different MRI scanners and field strengths [55, 102]. However, subcortical segmentations have been shown to have high reliability for thalamic measurements, low reliability for amygdala and intermediate reliability for hippocampus [103], especially when hippocampal abnormalities were present [104]. As described earlier in the method section, all brain images were manually inspected and structures with obvious segmentation errors were rejected. In order to avoid introducing bias and increasing variances into the data set of MRI images, no manual corrections were made. We used both questionnaires and a semi-structured diagnostic interview conducted by senior clinicians blinded to group adherence in order to identify psychiatric symptoms and disorders, allowing a thorough psychiatric evaluation. An experienced neuropsychologist performed all the IQ assessments at 19-years. US norms of the WAIS-III instead of Norwegian norms were used. Studies have shown that US norms are valid for Norwegian (and other Western Europe) samples with minor differences in mean subtask scores [105]. These possible differences would influence both study groups in the same way.

The participation rate was comparable to other follow-up studies with similar study groups [106] and participants and non-participants did not differ in socio-economic status or in perinatal variables (gestational age, birth weight, maternal age at birth), making selection bias less likely. Due to the relatively small sample of this study, only large differences and strong associations could reach significant levels. We had longitudinal data for a smaller sample than the cross-sectional study groups, which reduced the statistical power and thus, the generalization of the longitudinal results. In order to confirm our findings, studies with larger samples are definitely needed. However, the absolute volume differences between the VLBW subgroups and the control group, as well as the associations between symptoms and GM volumes in the VLBW subgroups were generally large as indicated by the low *p*-values, and hence unlikely to be due to chance.

## Conclusions

Our results indicate that significantly smaller subcortical GM volumes in VLBW adolescents compared with term-born peers might pose a risk for developing and maintaining psychiatric diagnoses during adolescence, and that extensive volume reductions affecting the thalamus, subcortical GM and occipital and parietal cortex might help to explain the higher rates of psychiatric symptoms found in VLBW adolescents. Future research should explore the possible role of reduced cortical and subcortical GM volumes in the pathogenesis of psychiatric illness in VLBW adolescents.

## Additional files

Additional file 1: Supplementary figure. Brain volumes in VLBW adolescents according to diagnostic status and controls at 15 and 19 years of age. In general, VLBW adolescents had smaller gray matter volumes than controls in cortical and subcortical areas at both 15 and 19 years (A-I). VLBW adolescents with psychiatric diagnosis had smaller cortical gray matter volumes than healthy VLBW adolescents at 15 years, but these differences disappeared at 19 years (A-G). There were not differences in thalamic volume and subcortical gray matter volume between the VLBW subgroups at 15 years. At 19 years, the healthy VLBW group had larger thalamic volumes than the VLBW group with subclinical diagnosis, and larger subcortical gray matter volume than the VLBW group with diagnosis. (TIF 773 kb)
Additional file 2: Appendix 1A. Brain volume (ml) differences between the two VLBW diagnostic groups and controls at 15 and 19 years of age. The two VLBW groups tended to have smaller brain volumes than the controls in all studied areas. Subcortical gray matter, was in the persisting/developing diagnosis VLBW group smaller than in both controls and the healthy/becoming healthy VLBW group. (DOCX 23 kb)
Additional file 3: Appendix 1B. Brain volume (ml) differences between the two VLBW diagnostic groups and controls at 15 and 19 years of age corrected for IQ. Both VLBW subgroups had persistent smaller volume of parietal cortex than controls at both time points. The healthy/becoming healthy VLBW subgroup had smaller cingulate and temporal volumes at both ages and smaller frontal cortical volume at 19 years than the control group. Thalamic volumes were smaller in the VLBW subgroup with persisting/developing diagnosis than in controls at both ages. (DOCX 22 kb)
Additional file 4: Appendix 2A. Brain growth differences between the two VLBW subgroups and the control group from 15 to 19 years of age. There were no differences in GM volume growth rate in the brain cortex, thalamus and subcortical GM between the two VLBW subgroups and controls. (DOCX 13 kb)
Additional file 5: Appendix 2B. Brain growth differences between the two VLBW diagnostic groups and the control group from 15 to 19 years of age corrected for IQ. There were no differences in GM volume growth rate in the brain cortex, thalamus and subcortical GM between the two VLBW subgroups and controls. (DOCX 13 kb)
Additional file 6: Appendix 3A. Relationship between brain volumes and psychiatric symptoms assessed with questionnaires in the VLBW group at 15 and 19 years of age. At 15 years of age, smaller volumes of occipital and parietal cortex and of thalamus predicted lower scores in general psychosocial functioning (CGAS scores). Smaller subcortical GM volumes predicted lower psychosocial functioning at both 15 and 19 years. Smaller volumes of occipital and parietal cortex predicted higher inattention scores in at both ages, although not all differences survived corrections for multiple comparisons. (DOCX 16 kb)
Additional file 7: Appendix 3B. Relationship between brain volumes and psychiatric symptoms assessed with questionnaires in the VLBW group at 15 and 19 years of age corrected for IQ. Occipital and parietal cortex volumes predicted lower scores in general psychosocial functioning at 15 years. Smaller volumes of occipital and parietal cortex predicted higher hyperactivity scores at 15 years. (DOCX 23 kb)
Additional file 8: Appendix 4A. Mixed linear regressions with psychiatric data as dependent variable and brain volumes (ml) and time as independent variables in the VLBW group. Adjusted for sex and total intracranial volume, but not for IQ. There were no associations between GM volume growth rate in the brain cortex, thalamus or in subcortical GM and CGAS scores in the VLBW group. (DOCX 19 kb)
Additional file 9: Appendix 4B. Mixed linear regressions with psychiatric data as dependent variable and brain volumes (ml) and time as independent variables in the VLBW group. Adjusted for sex, total intracranial volume and IQ. There were no associations between GM volume growth rate in the brain cortex, thalamus or in subcortical GM and CGAS scores in the VLBW group. (DOCX 18 kb)

## Abbreviations

ADHD-RS-IV: ADHD Rating Scale-IV
CGAS: Children’s Global Assessment Scale
GM: Gray matter
VLBW: Very low birth weight
VPT: Very preterm
WM: White matter
