# Supplementary figures and images for: A longitudinal study of associations between psychiatric symptoms and disorders and cerebral gray matter volumes in adolescents born very preterm
Source: BMC Pediatr. 2017 Feb 1;17:45. doi: 10.1186/s12887-017-0793-0 (PMC5286868; doi:10.1186/s12887-017-0793-0)

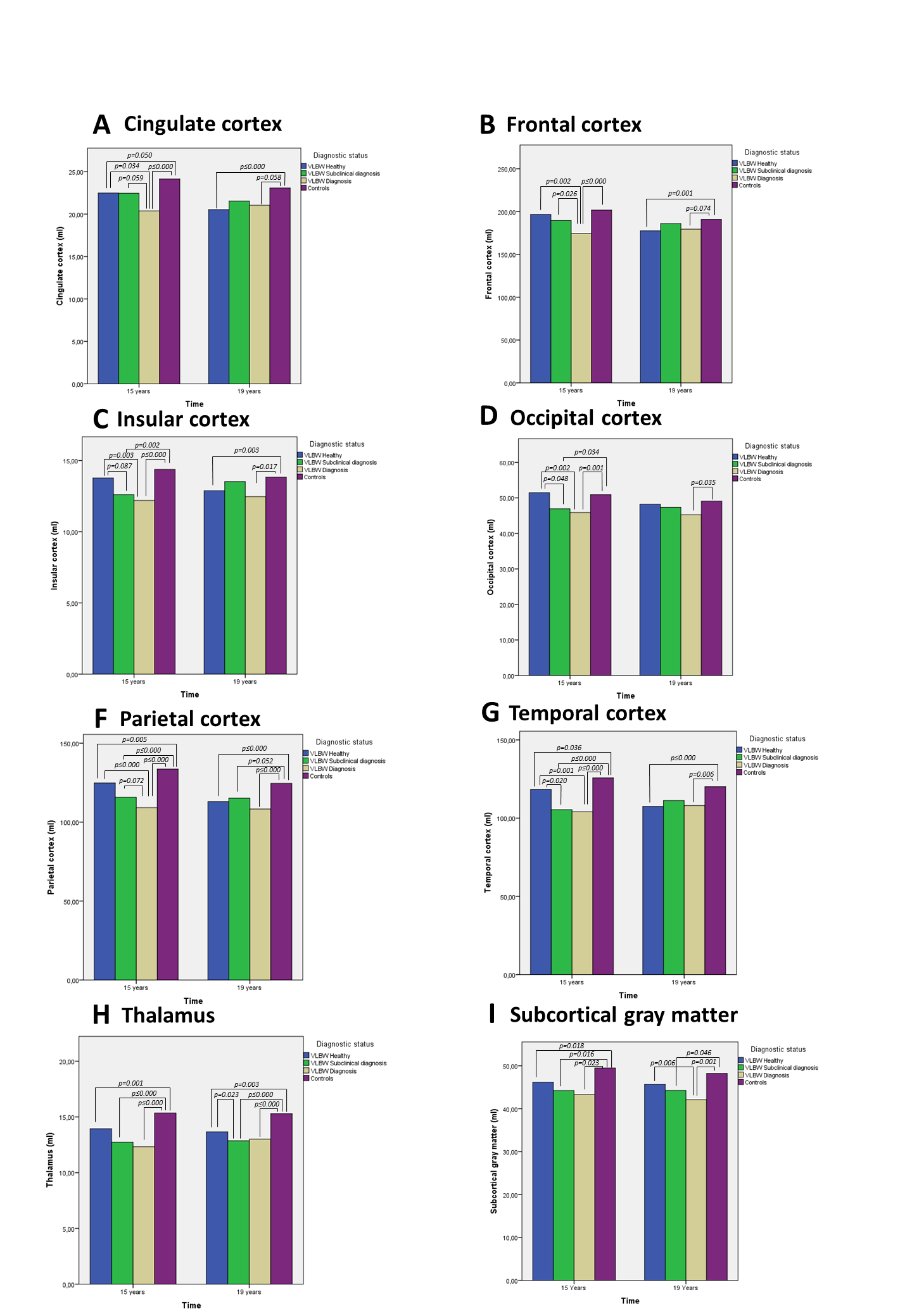

Supplement: Additional file 1: — Supplementary figure. Brain volumes in VLBW adolescents according to diagnostic status and controls at 15 and 19 years of age. In general, VLBW adolescents had smaller gray matter volumes than controls in cortical and subcortical areas at both 15 and 19 years (A-I). VLBW adolescents with psychiatric diagnosis had smaller cortical gray matter volumes than healthy VLBW adolescents at 15 years, but these differences disappeared at 19 years (A-G). There were not differences in thalamic volume and subcortical gray matter volume between the VLBW subgroups at 15 years. At 19 years, the healthy VLBW group had larger thalamic volumes than the VLBW group with subclinical diagnosis, and larger subcortical gray matter volume than the VLBW group with diagnosis. (TIF 773 kb) [file 12887_2017_793_MOESM1_ESM.tif]
