# Supplementary material for: A longitudinal study of associations between psychiatric symptoms and disorders and cerebral gray matter volumes in adolescents born very preterm
Source: BMC Pediatr. 2017 Feb 1;17:45. doi: 10.1186/s12887-017-0793-0 (PMC5286868; doi:10.1186/s12887-017-0793-0)
Supplement: Additional file 2: — Appendix 1A. Brain volume (ml) differences between the two VLBW diagnostic groups and controls at 15 and 19 years of age. The two VLBW groups tended to have smaller brain volumes than the controls in all studied areas. Subcortical gray matter, was in the persisting/developing diagnosis VLBW group smaller than in both controls and the healthy/becoming healthy VLBW group. (DOCX 23 kb) [file 12887_2017_793_MOESM2_ESM.docx]

| **Appendix 1A.** Brain volume (ml) differences between the two VLBW diagnostic groups and controls at 15 and 19 years of age. | | | | | | | | | | |
| --- | --- | --- | --- | --- | --- | --- | --- | --- | --- | --- |
|  | | **MD** | **SE** | | **(95% ci)** | ***p*-value** | **MD** | **SE** | **(95% ci)** | ***p*-value** |
|  | | **15 years** | | | | | **19 years** | | | |
| **Cortical gray matter** | |  |  |  | |  |  |  |  |  |
| **Cingulum** | |  |  |  | |  |  |  |  |  |
| Persisting/Developing diagnosis | Healthy/Becoming healthy | 0.047 | 0.952 | (-1.845 to 1.938) | | 0.961 | 0.959 | 0.864 | (-0.756 to 2.647) | 0.270 |
|  | Controls | -2.188 | 0.800 | (-3.778 to 0.598) | | **0.008*** | -1.651 | 0.762 | (-3.163 to -0.140) | **0.033** |
| Healthy/Becoming healthy | Controls | -2.234 | 0.775 | (-3.774 to 0.695) | | **0.005*** | -2.610 | 0.638 | (-3.877 to -1.343) | **≤0.001*** |
| **Frontal cortex** | |  |  |  | |  |  |  |  |  |
| Persisting/Developing diagnosis | Healthy/Becoming healthy | 3.546 | 6.934 | (-10.228 to 17.320) | | 0.610 | 5.237 | 5.669 | (-6.017 to 16.490) | 0.358 |
|  | Controls | -12.710 | 5.828 | (-24.286 to -1.134) | | **0.032** | -8.337 | 4.998 | (-18.757 to 1.083) | **0.080** |
| Healthy/Becoming healthy | Controls | -16.256 | 5.643 | (-27.464 to -5.048) | | **0.005*** | -14.074 | 4.188 | (-22.386 to -5.761) | **0.001*** |
| **Insula** | |  |  |  | |  |  |  |  |  |
| Persisting/Developing diagnosis | Healthy/Becoming healthy | -0.684 | 0.509 | (-1.695 to 0.327) | | 0.182 | 0.149 | 0.450 | (-0.743 to 1.042) | 0.741 |
|  | Controls | -1.735 | 0.428 | (-2.285 to -0.886) | | **≤0.001*** | -0.864 | 0.396 | (-1.651 to -0.077) | **0.032** |
| Healthy/Becoming healthy | Controls | -1.051 | 0.414 | (-1.874 to -0.229) | | **0.013** | -1.013 | 0.332 | (-1.672 to -0.354) | **0.003*** |
| **Occipital cortex** | |  |  |  | |  |  |  |  |  |
| Persisting/Developing diagnosis | Healthy/Becoming healthy | -1.671 | 1.698 | (-3.043 to 1.702) | | 0.328 | -1.778 | 1.495 | (-4.746 to 1.189) | 0.237 |
|  | Controls | -3.388 | 1.427 | (-6.222 to 0.554) | | **0.020** | -2.801 | 1.318 | 8-5.417 to -0.184) | **0.036** |
| Healthy/Becoming healthy | Controls | -1.717 | 1.382 | (-4.461 to 1.027) | | 0.217 | -1.022 | 1.104 | (-3.214 to 1.170) | 0.357 |
| **Parietal cortex** | |  |  |  | |  |  |  |  |  |
| Persisting/Developing diagnosis | Healthy/Becoming healthy | -5.711 | 4.264 | (-14.180 to 2.758) | | 0.184 | -1.435 | 3.297 | (-7.979 to 5.109) | 0.664 |
|  | Controls | -19.724 | 3.583 | (-26.841 to -12.606) | | **≤0.001*** | -13.436 | 2.906 | (-19.205 to -7.667) | **≤0.001*** |
| Healthy/Becoming healthy | Controls | -14.012 | 3.469 | (-20.904 to -7.121) | | **≤0.001*** | -12.001 | 2.435 | (-16.835 to -7.167) | **≤0.001*** |
| **Temporal cortex** | |  |  |  | |  |  |  |  |  |
| Persisting/Developing diagnosis | Healthy/Becoming healthy | 0.558 | 4.333 | (-8.049 to 9.164) | | 0.898 | 2.878 | 3.737 | (-4.540 to 10.297) | 0.443 |
|  | Controls | -14.847 | 3.641 | (-22.080 to -7.614) | | **≤0.001*** | -10.230 | 3.295 | (-16.770 to -3.691) | **0.002*** |
| Healthy/Becoming healthy | Controls | -15.405 | 3.526 | (-22.408 to -8.401) | | **≤0.001*** | -13.109 | 2.761 | (-18.589 to -7.629) | **≤0.001*** |
| **Thalamus** | |  |  |  | |  |  |  |  |  |
| Persisting/Developing diagnosis | Healthy/Becoming healthy | -1.239 | 0.363 | (-1.961 to -0.517) | | **0.001*** | -0.690 | 0.325 | (-1.334 to -0.045) | **0.036** |
|  | Controls | -2.068 | 0.330 | (-2.723 to -1.413) | | **≤0.001*** | -1.516 | 0.300 | (-2.112 to -0.920) | **≤0.001*** |
| Healthy/Becoming healthy | Controls | -0.829 | 0.317 | (-1.459 to -0.200) | | **0.010*** | -0.826 | 0.266 | (-1.355 to -0.297) | **0.003*** |
| **Subcortical gray matter** | |  |  |  | |  |  |  |  |  |
| Persisting/Developing diagnosis | Healthy/Becoming healthy | -3.820 | 1.045 | (-5.895 to -1.744) | | **≤0.001*** | -2.731 | 0.926 | (-4.569 to -0.893) | **0.004*** |
|  | Controls | -4.719 | 0.948 | (-6.602 to -2.837) | | **≤0.001*** | -3.213 | 0.856 | (-4.913 to -1.513) | **≤0.001*** |
| Healthy/Becoming healthy | Controls | -0.900 | 0.911 | (-2.709 to 0.910) | | 0.326 | -0.482 | 0.760 | (-1.991 to 1.027) | 0.528 |
| General Linear Model brain volumes (ml) as dependent variable and group as categorical independent variable at both time points. Adjusted for age, sex and total intracranial volume, but not IQ.  Significant results and trends marked bold. *****Significant results corrected for multiple comparisons using the Benjamini-Hochberg procedure.  *Abbreviations*: ci: Confidence interval; IQ: Intelligence Quotient; MD: Mean difference; SE: Standard error; VLBW: Very low birth weight. | | | | | | | | | | |
