# Supplementary material for: A longitudinal study of associations between psychiatric symptoms and disorders and cerebral gray matter volumes in adolescents born very preterm
Source: BMC Pediatr. 2017 Feb 1;17:45. doi: 10.1186/s12887-017-0793-0 (PMC5286868; doi:10.1186/s12887-017-0793-0)
Supplement: Additional file 3: — Appendix 1B. Brain volume (ml) differences between the two VLBW diagnostic groups and controls at 15 and 19 years of age corrected for IQ. Both VLBW subgroups had persistent smaller volume of parietal cortex than controls at both time points. The healthy/becoming healthy VLBW subgroup had smaller cingulate and temporal volumes at both ages and smaller frontal cortical volume at 19 years than the control group. Thalamic volumes were smaller in the VLBW subgroup with persisting/developing diagnosis than in controls at both ages. (DOCX 22 kb) [file 12887_2017_793_MOESM3_ESM.docx]

| **Appendix 1B.** Brain volume (ml) differences between the two VLBW diagnostic groups and controls at 15 and 19 years of age corrected for IQ. | | | | | | | | | | |
| --- | --- | --- | --- | --- | --- | --- | --- | --- | --- | --- |
|  | | **MD** | **SE** | | **(95% ci)** | ***p*-value** | **MD** | **SE** | **(95% ci)** | ***p*-value** |
|  | | **15 years** | | | | | **19 years** | | | |
| **Cortical gray matter** | |  |  |  | |  |  |  |  |  |
| **Cingulum** | |  |  |  | |  |  |  |  |  |
| Persisting/Developing diagnosis | Healthy/Becoming healthy | 0.950 | 1.203 | (-1.455 to 3.355) | | 0.433 | 1.127 | 0.886 | (-4.736 to 16.041) | 0.207 |
|  | Controls | -2.023 | 1.217 | (-4.456 to 0.410) | | 0.102 | -1.328 | 0.848 | (-3.011 to 0.355) | 0.121 |
| Healthy/Becoming healthy | Controls | -2.973 | 0.929 | (-4.830 to -1.116) | | **0.002*** | -2.455 | 0.663 | (-3.772 to -1.139) | **≤0.001*** |
| **Frontal cortex** | |  |  |  | |  |  |  |  |  |
| Persisting/Developing diagnosis | Healthy/Becoming healthy | 9.045 | 9.092 | (-9.135 to 27.225) | | 0.324 | 6.713 | 5.798 | (-4.796 to 18.223) | 0.250 |
|  | Controls | -8.948 | 9.199 | (-27.343 to 9.446) | | 0.335 | -6.002 | 0.282 | (-17.012 to 5.007) | 0.282 |
| Healthy/Becoming healthy | Controls | -17.993 | 5.804 | (-32.032 to -3.954) | | **0.013** | -12.715 | 4.338 | (-21.328 to -4.103) | **0.004*** |
| **Insula** | |  |  |  | |  |  |  |  |  |
| Persisting/Developing diagnosis | Healthy/Becoming healthy | 0.021 | 0.667 | (-1.312 to 1.355) | | 0.974 | 0.327 | 0.455 | (-0.577 to 1.231) | 0.475 |
|  | Controls | -1.061 | 0.675 | (-2.410 to 0.288) | | 0.121 | -0.523 | 0.436 | (-1.388 to 0.342) | 0.233 |
| Healthy/Becoming healthy | Controls | -1.083 | 0.515 | (-2.112 to -0.053) | | **0.040** | -0.850 | 0.341 | (-1.526 to -0.173) | **0.014** |
| **Occipital cortex** | |  |  |  | |  |  |  |  |  |
| Persisting/Developing diagnosis | Healthy/Becoming healthy | -0.944 | 2.271 | (-5.285 to 3.397) | | 0.665 | -1.058 | 1.502 | (-4.040 to 1.923) | 0.483 |
|  | Controls | -1.768 | 2.196 | (-6.160 to 2.625) | | 0.424 | -1.418 | 1.437 | (-4.271 to -1.434) | 0.326 |
| Healthy/Becoming healthy | Controls | -0.823 | 1.380 | (-4.176 to 2.529) | | 0.625 | -0.360 | 1.124 | (-2.591 to 1.871) | 0.749 |
| **Parietal cortex** | |  |  |  | |  |  |  |  |  |
| Persisting/Developing diagnosis | Healthy/Becoming healthy | -3.296 | 5.642 | (-14.578 to 7.986) | | 0.561 | -0.802 | 3.382 | (-7.517 to 5.913) | 0.813 |
|  | Controls | -17.814 | 5.708 | (-29.229 to -6.399) | | **0.003*** | -12.220 | 3.235 | (-18.644 to -5.797) | **0.001*** |
| Healthy/Becoming healthy | Controls | -14.518 | 4.357 | (-23.230 to -5.806) | | **0.001*** | -11.418 | 2.531 | (-16.443 to -6.394) | **0.001*** |
| **Temporal cortex** | |  |  |  | |  |  |  |  |  |
| Persisting/Developing diagnosis | Healthy/Becoming healthy | 4.697 | 5.848 | (-6.996 to 16.390) | | 0.425 | 3.195 | 3.847 | (-4.442 to 10.831) | 0.408 |
|  | Controls | -12.472 | 5.916 | (-24.303 to -0.641) | | **0.039** | -9.623 | 3.679 | (-16.928 to -2.319) | **0.010** |
| Healthy/Becoming healthy | Controls | -17.169 | 4.515 | (-26.199 to -8.140) | | **≤0.001*** | -12.818 | 2.878 | (-18.532 to -7.104) | **≤0.001*** |
| **Thalamus** | |  |  |  | |  |  |  |  |  |
| Persisting/Developing diagnosis | Healthy/Becoming healthy | -0.794 | 0.432 | (-1.658 to 0.069) | | **0.071** | -0.524 | 0.326 | (-1.172 to 0.124) | 0.112 |
|  | Controls | -1.508 | 0.450 | (-2.408 to -0.609) | | **0.001*** | -1.229 | 0.320 | (-1.865 to -0.593) | **≤0.001*** |
| Healthy/Becoming healthy | Controls | -0.714 | 0.357 | (-1.428 to -0.001) | | **0.050** | -0.705 | 0.266 | (-1.234 to -0.176) | **0.010** |
| **Subcortical gray matter** | |  |  |  | |  |  |  |  |  |
| Persisting/Developing diagnosis | Healthy/Becoming healthy | -2.631 | 1.265 | (-5.161 to -0.101) | | **0.042** | -2.258 | 0.931 | (-4.106 to -0.410) | **0.017** |
|  | Controls | -2.764 | 1.317 | (-5.399 to -0.129) | | **0.040** | -2.394 | 0.914 | (-4.208 to -0.579) | **0.010** |
| Healthy/Becoming healthy | Controls | -0.132 | 1.045 | (-2.223 to 1.958) | | 0.900 | -0.136 | 0.760 | (-1.644 to 1.373) | 0.859 |
| General Linear Model brain volumes (ml) as dependent variable and group as categorical independent variable at both time points. Adjusted for age, sex and IQ. Subcortical volumes corrected for estimated intracranial volume.  Significant results and trends marked bold. *****Significant results corrected for multiple comparisons using the Benjamini-Hochberg procedure.  *Abbreviations*: ci: Confidence interval; IQ: Intelligence Quotient; MD: Mean difference; SE: Standard error; VLBW: Very low birth weight. | | | | | | | | | | |
