# Supplementary material for: A longitudinal study of associations between psychiatric symptoms and disorders and cerebral gray matter volumes in adolescents born very preterm
Source: BMC Pediatr. 2017 Feb 1;17:45. doi: 10.1186/s12887-017-0793-0 (PMC5286868; doi:10.1186/s12887-017-0793-0)
Supplement: Additional file 4: — Appendix 2A. Brain growth differences between the two VLBW subgroups and the control group from 15 to 19 years of age. There were no differences in GM volume growth rate in the brain cortex, thalamus and subcortical GM between the two VLBW subgroups and controls. (DOCX 13 kb) [file 12887_2017_793_MOESM4_ESM.docx]

| **Appendix 2A:**  Brain growth differences between the two VLBW subgroups and the control group from 15 to 19 years of age. | | | |
| --- | --- | --- | --- |
|  | **Interaction time x group** | | |
|  | ***Coefficient*** | ***(95% ci)*** | ***p-value*** |
| Cortical gray matter |  |  |  |
| Cingulum | -0.159 | (-0.904 to 0.586) | 0.675 |
| Frontal cortex | -1.297 | ( -9.948 to 7.354) | 0.769 |
| Insula | 0.107 | ( -0.338 to 0.552) | 0.637 |
| Occipital cortex | -0.595 | ( -2.651 to 1.461) | 0.571 |
| Parietal cortex | 1.057 | ( -5.366 to 7.480) | 0.747 |
| Temporal cortex | -2.146 | ( -6.030 to 1.737) | 0.279 |
| Thalamus | 0.129 | (-0.165 to 0.424) | 0.389 |
| Subcortical gray matter | 0.020 | ( -0.690 to 0.730) | 0.955 |
| Mixed linear regressions with groups and time as independent variables and brain volumes (ml) as dependent variable. Adjusted for sex and IQ. Subcortical structures adjusted for estimated intracranial volume.  *Abbreviations*: ci: confidence interval; IQ: Intelligence Quotient; VLBW: Very low birth weight. | | | |
