# Supplementary material for: A longitudinal study of associations between psychiatric symptoms and disorders and cerebral gray matter volumes in adolescents born very preterm
Source: BMC Pediatr. 2017 Feb 1;17:45. doi: 10.1186/s12887-017-0793-0 (PMC5286868; doi:10.1186/s12887-017-0793-0)
Supplement: Additional file 5: — Appendix 2B. Brain growth differences between the two VLBW diagnostic groups and the control group from 15 to 19 years of age corrected for IQ. There were no differences in GM volume growth rate in the brain cortex, thalamus and subcortical GM between the two VLBW subgroups and controls. (DOCX 13 kb) [file 12887_2017_793_MOESM5_ESM.docx]

| **Appendix 2B:**  Brain growth differences between the two VLBW subgroups and the control group from 15 to 19 years of age corrected for IQ. | | | |
| --- | --- | --- | --- |
|  | **Interaction time x group** | | |
|  | ***Coefficient*** | ***(95% ci)*** | ***p-value*** |
| Cortical gray matter |  |  |  |
| Cingulum | -0.006 | (-0.231 to 0.219) | 0.959 |
| Frontal cortex | 0.035 | ( -2.504 to 2.575) | 0.978 |
| Insula | -0.075 | ( -0.204 to 0.054) | 0.254 |
| Occipital cortex | -0.084 | ( -0.690 to 0.521) | 0.785 |
| Parietal cortex | -0.081 | ( -2.043 to 1.881) | 0.936 |
| Temporal cortex | -0.645 | ( -1.800 to 0.510) | 0.274 |
| Thalamus | 0.075 | ( -0.049to 0.200) | 0.236 |
| Subcortical gray matter | 0.111 | ( -0.152 to 0.373) | 0.408 |
| Mixed linear regressions with groups and time as independent variables and brain volumes (ml) as dependent variable. Adjusted for sex and IQ. Subcortical structures adjusted for estimated intracranial volume.  *Abbreviations*: ci: confidence interval; IQ: Intelligence Quotient; VLBW: Very low birth weight. | | | |
