# Supplementary material for: A longitudinal study of associations between psychiatric symptoms and disorders and cerebral gray matter volumes in adolescents born very preterm
Source: BMC Pediatr. 2017 Feb 1;17:45. doi: 10.1186/s12887-017-0793-0 (PMC5286868; doi:10.1186/s12887-017-0793-0)
Supplement: Additional file 6: — Appendix 3A. Relationship between brain volumes and psychiatric symptoms assessed with questionnaires in the VLBW group at 15 and 19 years of age. At 15 years of age, smaller volumes of occipital and parietal cortex and of thalamus predicted lower scores in general psychosocial functioning (CGAS scores). Smaller subcortical GM volumes predicted lower psychosocial functioning at both 15 and 19 years. Smaller volumes of occipital and parietal cortex predicted higher inattention scores in at both ages, although not all differences survived corrections for multiple comparisons. (DOCX 16 kb) [file 12887_2017_793_MOESM6_ESM.docx]

| **Appendix 3A:**  Relationship between brain volumes and psychiatric symptoms assessed with questionnaires in the VLBW group at 15 and 19 years of age. | | | | | | |
| --- | --- | --- | --- | --- | --- | --- |
|  | **15 years** | | | **19 years** | | |
|  | ***Coefficient*** | ***(95% ci)*** | ***p-value*** | ***Coefficient*** | ***(95% ci)*** | ***p-value*** |
| ***CGAS*** *(T1n*=*40, T2 n=41)* |  |  |  |  |  |  |
| Cortical gray matter |  |  |  |  |  |  |
| Cingulum | 0.730 | (-0.748 to 2.207) | 0.323 | -0.911 | (-2.333 to 0.510) | 0.202 |
| Frontal cortex | 0.124 | (-0.035 to -0.283) | 0.122 | -0.065 | (-0.254 to 0.124) | 0.492 |
| Insula | 2.311 | (0.121 to 4.502) | **0.039** | -0.189 | (-2.575 to 2.196) | 0.873 |
| Occipital cortex | 1.107 | (0.465 to 1.750) | **0.001*** | 0.273 | (-0.445 to 0.990) | 0.446 |
| Parietal cortex | 0.366 | (0.109 to 0.622) | **0.007*** | 0.103 | (-0.235 to 0.440) | 0.541 |
| Temporal cortex | 0.245 | (-0.023 to 0.514) | **0.072** | -0.088 | (-0.391 to 0.216) | 0.562 |
| Thalamus | 3.990 | (1.457 to 6.523) | **0.003*** | 2.909 | (-0.641 to 6.459) | 0.105 |
| Subcortical gray matter | 1.441 | (0.505 to 2.377) | **0.004*** | 1.454 | (0.391 to 2.517) | **0.009*** |
| **ADHD-RS mother-report** *(T1 n*=36, *T2 n=29)* | |  |  |  |  |  |
| **Hyperactivity** |  |  |  |  |  |  |
| Cortical gray matter |  |  |  |  |  |  |
| Cingulum | -0.293 | (-0.991 to 0.405) | 0.397 | -0.079 | (-0.792 to 0.694) | 0.792 |
| Frontal cortex | -0.031 | (-0.108 to 0.045) | 0.407 | 0.001 | (-0.080 to 0.081) | 0.990 |
| Insula | -0.385 | (-1.473 to 0.703) | 0.474 | -0.238 | (-0.768 to 1.224) | 0.630 |
| Occipital cortex | -0.215 | (-0.555 to 0.126) | 0.207 | -0.243 | (-0.488 to 0.003) | **0.053** |
| Parietal cortex | -0.046 | (-0.180 to 0.087) | 0.483 | -0.134 | -0.246 to -0.021) | **0.021** |
| Temporal cortex | -0.020 | (-0.153 to 0.112) | 0.755 | 0.009 | (-0.120 to 0.139) | 0.883 |
| Thalamus | -0.051 | (-0.735 to 0.837) | 0.895 | -0.089 | (-1.358 to 1.180) | 0.887 |
| Subcortical gray matter | -0.120 | (-0.406 to 0.165) | 0.397 | -0.068 | (-0.567 to 0.431) | 0.781 |
| **Inattention** |  |  |  |  |  |  |
| Cortical gray matter |  |  |  |  |  |  |
| Cingulum | -0.409 | (-0.932 to 0.114) | 0.121 | -0.173 | (-0.999 to 0.653) | 0.669 |
| Frontal cortex | -0.018 | (-0.087 to 0.051) | 0.604 | -0.033 | (-0.140 to 0.075) | 0.535 |
| Insula | -0.631 | (-1.443 to 0.182) | 0.124 | -0.190 | (-1.549 to 1.168) | 0.775 |
| Occipital cortex | -0.356 | (-0.593 to -0.119) | **0.004*** | -0.408 | (-0.689 to -0.127) | **0.006*** |
| Parietal cortex | -0.105 | (-0.205 to 0.014) | **0.026** | -0.202 | (-0.331 to -0.072) | **0.003*** |
| Temporal cortex | -0.075 | (-0.174 to 0.023) | 0.128 | -0.021 | (-0.195 to 0.153) | 0.803 |
| Thalamus | -0.860 | (-1.882 to 0.162) | **0.096** | -1.166 | (-2.624 to 0.292) | 0.113 |
| Subcortical gray matter | -0.375 | (-0.744 to -0.007) | **0.046** | -0.432 | (-1.072 to 0.207) | 0.175 |
| Linear regressions with psychiatric data as dependent variable and brain volumes (ml) as independent variable in the VLBW group at both time points. Adjusted for age and sex, but not for IQ. Subcortical volumes corrected for estimated intracranial volume.  Significant results marked bold. ***** Significant results corrected for multiple comparisons using the Benjamini-Hochberg procedure.  *Abbreviations*: ADHD-RS: Attention Deficit Hyperactivity Disorder Rating Scale; CGAS: Children’s Global Assessment Scale; ci: confidence interval; IQ: Intelligence Quotient; VLBW: Very low birth weight. | | | | | | |
