# Supplementary material for: A longitudinal study of associations between psychiatric symptoms and disorders and cerebral gray matter volumes in adolescents born very preterm
Source: BMC Pediatr. 2017 Feb 1;17:45. doi: 10.1186/s12887-017-0793-0 (PMC5286868; doi:10.1186/s12887-017-0793-0)
Supplement: Additional file 7: — Appendix 3B. Relationship between brain volumes and psychiatric symptoms assessed with questionnaires in the VLBW group at 15 and 19 years of age corrected for IQ. Occipital and parietal cortex volumes predicted lower scores in general psychosocial functioning at 15 years. Smaller volumes of occipital and parietal cortex predicted higher hyperactivity scores at 15 years. (DOCX 23 kb) [file 12887_2017_793_MOESM7_ESM.docx]

| **Appendix 3B:**  Relationship between brain volumes and psychiatric symptoms assessed with questionnaires in the VLBW group at 15 and 19 years of age corrected for IQ. | | | | | | |
| --- | --- | --- | --- | --- | --- | --- |
|  | **15 years** | | | **19 years** | | |
|  | ***Coefficient*** | ***(95% ci)*** | ***p-value*** | ***Coefficient*** | ***(95% ci)*** | ***p-value*** |
| ***CGAS*** *(T1n*=*40, T2 n=41)* |  |  |  |  |  |  |
| Cortical gray matter |  |  |  |  |  |  |
| Cingulum | 0.188 | (-1.191to 2.207) | 0.781 | -1.234 | (-2.447 to 0.009) | **0.052** |
| Frontal cortex | 0.052 | (-0.099 to 0.204) | 0.483 | -0.152 | (-0.321 to 0.017) | **0.077** |
| Insula | 0.883 | (-1.321 to 3.086) | 0.417 | -1.384 | (-3.577 to 0.809) | 0.209 |
| Occipital cortex | 0.613 | (0.001 to 1.224) | **0.004*** | -0.458 | (-1.225 to 0.310) | 0.234 |
| Parietal cortex | 0.246 | (0.031 to 0.461) | **0.006*** | -0.104 | (-0.431 to 0.224) | 0.524 |
| Temporal cortex | 0.220 | (-0.019 to 0.458) | **0.069** | -0.123 | (-0.393 to .147) | 0.362 |
| Thalamus | 1.516 | (-1.738 to 4.770) | 0.346 | 2.909 | (-0.641 to 6.459) | 0.105 |
| Subcortical gray matter | 0.418 | (-0.791 to 1.627) | 0.483 | 0.624 | (-0.596 to 1.845) | 0.306 |
| **ADHD-RS mother-report** *(T1 n*=36, *T2 n=29)* | |  |  |  |  |  |
| **Hyperactivity** |  |  |  |  |  |  |
| Cortical gray matter |  |  |  |  |  |  |
| Cingulum | -0.460 | (-0.906 to 0.013) | 0.044 | -0.229 | (-0.747 to 0.2889 | 0.373 |
| Frontal cortex | -0.056 | (-0.105 to -0.008) | **0.025** | -0.043 | (-0.121 to 0.026) | 0.215 |
| Insula | -0.610 | (-1.353 to 0.133) | 0.103 | -0.456 | (-1.337 to 0.425) | 0.299 |
| Occipital cortex | -0.363 | (-0.597 to -0.129) | **0.004*** | -0.214 | (-0.517 to 0.089) | 0.159 |
| Parietal cortex | -0.105 | (-0.178 to -0.033) | **0.006*** | -0.121 | (-0.245 to 0.003) | **0.056** |
| Temporal cortex | -0.084 | (-0.159 to -0.009) | **0.029** | -0.057 | (-0.164 to 0.051) | 0.289 |
| Thalamus | 0.428 | (-0.650 to 1.505) | 0.420 | -0.089 | (-1.358 to 1.180) | 0.887 |
| Subcortical gray matter | 0.068 | (-0.333 to 0.470) | 0.728 | -0.231 | (-0.718 to 0.256) | 0.340 |
| **Inattention** |  |  |  |  |  |  |
| Cortical gray matter |  |  |  |  |  |  |
| Cingulum | -0.113 | (-0.888 to 0.663) | 0.764 | -0.286 | (-0.864 to 0.293) | 0.321 |
| Frontal cortex | -0.030 | (-0.092 to 0.032) | 0.329 | -0.046 | (-0.123 to 0.032) | 0.235 |
| Insula | -0.308 | (-1.228 to 0.612) | 0.496 | -0.464 | 8-1.456 to 0.527) | 0.346 |
| Occipital cortex | -0.242 | (-0.556 to -0.073) | 0.126 | -0.277 | (-0.612 to 0.059) | 0.103 |
| Parietal cortex | -0.074 | (-0.183 to 0.036) | 0.179 | -0.154 | (-0.290 to -0.017) | **0.029** |
| Temporal cortex | -0.075 | (-0.174 to 0.023) | 0.128 | -0.087 | (-0.205 to 0.032) | 0.146 |
| Thalamus | -0.305 | (-1.692 to 1.081) | 0.653 | -1.166 | (-2.624 to 0.292) | 0.113 |
| Subcortical gray matter | -0.139 | (-0.649 to 0.370) | 0.577 | -0.317 | (-0.858 to 0.225) | 0.225 |
| Linear regressions with psychiatric data as dependent variable and brain volumes (ml) as independent variable in the VLBW group at both time points. Adjusted for age, sex and IQ. Subcortical volumes corrected for estimated intracranial volume.  Significant results and trends marked bold. ***** Significant results corrected for multiple comparisons using the Benjamini-Hochberg procedure.  *Abbreviations*: ADHD-RS: Attention Deficit Hyperactivity Disorder Rating Scale; CGAS: Children’s Global Assessment Scale; ci: confidence interval; IQ: Intelligence Quotient; VLBW: Very low birth weight. | | | | | | |
