# Supplementary material for: A longitudinal study of associations between psychiatric symptoms and disorders and cerebral gray matter volumes in adolescents born very preterm
Source: BMC Pediatr. 2017 Feb 1;17:45. doi: 10.1186/s12887-017-0793-0 (PMC5286868; doi:10.1186/s12887-017-0793-0)
Supplement: Additional file 8: — Appendix 4A. Mixed linear regressions with psychiatric data as dependent variable and brain volumes (ml) and time as independent variables in the VLBW group. Adjusted for sex and total intracranial volume, but not for IQ. There were no associations between GM volume growth rate in the brain cortex, thalamus or in subcortical GM and CGAS scores in the VLBW group. (DOCX 19 kb) [file 12887_2017_793_MOESM8_ESM.docx]

| **Appendix 4A:** Mixed linear regressions with psychiatric data as dependent variable and brain volumes (ml) and time as independent variables in the VLBW group. Adjusted for sex and total intracranial volume, but not for IQ. | | | |
| --- | --- | --- | --- |
|  | **Interaction time x brain** | | |
|  | ***Coefficient*** | ***(95% ci)*** | ***p-value*** |
| ***CGAS*** *(T1n*=*40, T2 n=41)* |  |  |  |
| Cortical gray matter |  |  |  |
| Cingulum | -0.888 | (-2.088 to 0.313) | 0.147 |
| Frontal cortex | -0.133 | (-0.273 to 0.008) | 0.064 |
| Insula | -1.200 | ( -2.955 to 0.554) | 0.180 |
| Occipital cortex | -0.461 | (-1.045 to 0.123) | 0.122 |
| Parietal cortex | -0.147 | 8 -.419 to0.125) | 0.290 |
| Temporal cortex | -0.195 | ( -0.405 to 0.016) | 0.070 |
| Thalamus | -1.655 | (-3.875 to 0.565) | 0.144 |
| Subcortical gray matter | -0.330 | ( -1.276 to 0.617) | 0.495 |
| **ADHD-RS mother-report** *(T1 n*=36, *T2 n=29)* | |  |  |
| **Hyperactivity** |  |  |  |
| Cortical gray matter |  |  |  |
| Cingulum | -0.065 | ( -.873 to 0.743) | 0.875 |
| Frontal cortex | -0.012 | (-0.092 to 0.068) | 0.773 |
| Insula | -0.090 | ( -1.031 to 0.852) | 0.852 |
| Occipital cortex | 0.046 | (-.294 to 0.386) | 0.791 |
| Parietal cortex | -0.055 | (-0.196 to 0.086) | 0.447 |
| Temporal cortex | -0.029 | ( -0.145 to 0.087) | 0.625 |
| Thalamus | 0.151 | (-1.036 to 1.338) | 0.803 |
| Subcortical gray matter | -0.330 | ( -1.276to 0.617) | 0.495 |
| **Inattention** |  |  |  |
| Cortical gray matter |  |  |  |
| Cingulum | -0.216 | ( -1.157 to 0.726) | 0.653 |
| Frontal cortex | -0.028 | ( -0.122 to 0.066) | 0.557 |
| Insula | -0.258 | ( -1.394 to 0.878) | 0.656 |
| Occipital cortex | -0.048 | (-0.419 to 0.323) | 0.799 |
| Parietal cortex | -0.077 | ( -0.233 to 0.078) | 0.330 |
| Temporal cortex | -0.038 | ( -0.178 to 0.103) | 0.598 |
| Thalamus | -0.362 | (-1.684 to 0.960) | 0.592 |
| Subcortical gray matter | -0.228 | ( -0.787 to 0.331) | 0.424 |
| Adjusted for sex, but not for IQ. Subcortical structures adjusted for estimated intracranial volume.  *Abbreviations*: ADHD-RS: Attention Deficit Hyperactivity Disorder Rating Scale; CGAS: Children’s Global Assessment Scale; ci: confidence interval; IQ: Intelligence Quotient; VLBW: Very low birth weight. | | | |
