# Supplementary material for: A longitudinal study of associations between psychiatric symptoms and disorders and cerebral gray matter volumes in adolescents born very preterm
Source: BMC Pediatr. 2017 Feb 1;17:45. doi: 10.1186/s12887-017-0793-0 (PMC5286868; doi:10.1186/s12887-017-0793-0)
Supplement: Additional file 9: — Appendix 4B. Mixed linear regressions with psychiatric data as dependent variable and brain volumes (ml) and time as independent variables in the VLBW group. Adjusted for sex, total intracranial volume and IQ. There were no associations between GM volume growth rate in the brain cortex, thalamus or in subcortical GM and CGAS scores in the VLBW group. (DOCX 18 kb) [file 12887_2017_793_MOESM9_ESM.docx]

| **Appendix 4B:** Mixed linear regressions with psychiatric data as dependent variable and brain volumes (ml) and time as independent variables in the VLBW group. Adjusted for sex, total intracranial volume and IQ. | | | |
| --- | --- | --- | --- |
|  | **Interaction time x brain** | | |
|  | ***Coefficient*** | ***(95% ci)*** | ***p-value*** |
| ***CGAS*** *(T1n*=*40, T2 n=41)* |  |  |  |
| Cortical gray matter |  |  |  |
| Cingulum | -0.684 | (-1.971 to 0.604) | 0.298 |
| Frontal cortex | -0.113 | (-.262 to 0.036) | 0.138 |
| Insula | -0.535 | (-2.432 to 1.362) | 0.580 |
| Occipital cortex | -0.149 | (-0.796 to .0498) | 0.652 |
| Parietal cortex | -.068 | ( -.348 to .212) | 0.634 |
| Temporal cortex | -0.159 | (-0.386 to 0.069) | 0.171 |
| Thalamus | -0.769 | (-3.195to 1.658) | 0.535 |
| Subcortical gray matter | 0.376 | ( -0.635to 1.387) | 0.466 |
| **ADHD-RS mother-report** *(T1 n*=36, *T2 n=29)* | |  |  |
| **Hyperactivity** |  |  |  |
| Cortical gray matter |  |  |  |
| Cingulum | -0.066 | (-0.843 to 0.711) | 0.868 |
| Frontal cortex | 0.003 | ( -0.075 to 0.080) | 0.942 |
| Insula | -0.071 | ( -1.005 to 0.864) | 0.882 |
| Occipital cortex | -0.005 | (0.328 to 0.318) | 0.975 |
| Parietal cortex | -0.035 | (-.170 to 0.010) | 0.608 |
| Temporal cortex | -0.009 | (-0.124 to 0.106) | 0.877 |
| Thalamus | 0.085 | ( -1.065 to 1.235) | 0.885 |
| Subcortical gray matter | 0.376 | (-0.634 to 1.387) | 0.466 |
| **Inattention** |  |  |  |
| Cortical gray matter |  |  |  |
| Cingulum | -0.278 | (-1.180 to 0.624) | 0.546 |
| Frontal cortex | -0.020 | (0.110 to 0.070) | 0.658 |
| Insula | -0.295 | (-1.381 to 0.791) | 0.594 |
| Occipital cortex | -0.085 | ( -0.454 to 0.284) | 0.652 |
| Parietal cortex | -0.068 | (-0.220 to 0.085) | 0.383 |
| Temporal cortex | -0.031 | (-0.164 to 0.102) | 0.650 |
| Thalamus | -0.574 | (-1.907 to 0.758) | 0.398 |
| Subcortical gray matter | -0.369 | (-0.933 to 0.194) | 0.199 |
| Adjusted for sex and IQ. Subcortical structures adjusted for estimated intracranial volume.  *Abbreviations*: ADHD-RS: Attention Deficit Hyperactivity Disorder Rating Scale; CGAS: Children’s Global Assessment Scale; ci: confidence interval; IQ: Intelligence Quotient; VLBW: Very low birth weight. | | | |
